# Impact of Training Based on Orem's Theory on Self-Care Agency and Quality of Life in Patients With Coronary Artery Disease

**DOI:** 10.1097/JNR.0000000000000406

**Published:** 2020-10-05

**Authors:** Fatma TOK YILDIZ, Mağfiret KAŞIKÇI

**Affiliations:** 1 PhD, RN, Assistant Professor, Program of Anaesthesia, Department of Medical Services and Techniques, Vocational School of Health Services, Sivas Cumhuriyet University, Sivas, Turkey; 2 PhD, RN, Professor, Faculty of Nursing, Atatürk University, Erzurum, Turkey.

**Keywords:** training, coronary artery disease, Orem's self-care deficit nursing theory, self-care agency, quality of life

## Abstract

**Background:**

Coronary artery disease (CAD) is a primary cause of death worldwide. CAD negatively affects individuals because it reduces their functional skills and self-care abilities and disrupts quality of life.

**Purpose:**

This study was designed to assess the impact of a training program based on Orem's self-care deficit nursing theory (SCDNT) on self-care abilities and quality of life in patients with CAD.

**Methods:**

This study was conducted using a randomized, controlled, pretest/posttest experimental design. One hundred two patients with CAD were divided evenly into either the intervention or control group, with sample randomization based on gender, age, low-density lipoprotein cholesterol level, and Self-Care Agency Scale scores. For both groups, interviews were conducted in two sessions held, respectively, at the hospital and at home. Study data were collected using the patient information form, Self-Care Agency Scale, MacNew Heart Disease Health-Related Quality of Life Questionnaire (MacNew), Quality of Life Questionnaire (15D), and training booklet.

**Results:**

A highly significant difference was found between the two groups in terms of the average posttest scores on the Self-Care Agency Scale, MacNew, and 15D. For the intervention group, the posttest scores on the Self-Care Agency Scale, MacNew, and 15D were significantly higher than the pretest scores, whereas average pretest and posttest scores on these measures were similar for the control group.

**Conclusions:**

The training program developed in this study based on Orem's SCDNT improved self-care agency as well as disease-specific and overall quality of life in patients with CAD. Nurses should pay attention to the CAD-related educational level of patients when teaching them how to live with their disease. Moreover, nurses should use Orem's SCDNT to strengthen the self-care agency of these patients to increase quality of life and the effectiveness of related education efforts. Finally, medical institutions and governments should develop appropriate education policies for patients at risk of CAD and for those with CAD.

## Introduction

According to the global implementation plan of the World Health Organization (WHO) on preventable and controllable noncommunicable diseases, cardiovascular disease, cancer, chronic respiratory tract diseases, and diabetes are the primary causes of deaths worldwide, with incidences of 48%, 21%, 12%, and 3.5%, respectively (WHO, 2019). Cardiovascular disease is the leading cause of morbidity and mortality in Turkey. According to the 2009 Cardiac Diseases and Risk Factors in Turkish Adults report, coronary artery disease (CAD) is the largest cause of death in Turkey (Onat, 2009).

CAD negatively affects an individual's course of life, maintenance of health, and progression of disease owing to its accompanying physical, psychological, social, and economic problems (Hassani et al., 2010; Sevinç & Akyol, 2010). In addition, this disease reduces functional skills and self-care abilities (Hassani et al., 2010), prevents completion of self-care responsibilities, and disrupts quality of life (Dilek et al., 2010; Durmaz et al., 2009; Norris et al., 2009). Studies on CAD have identified a high prevalence of modifiable risk factors and determined that effective risk factor management may substantially reduce the pace of morbidity and mortality and, eventually, improve health and quality of life (Durmaz et al., 2009; Saffi et al., 2014; Sevinç & Akyol, 2010).

Education is crucial to increasing awareness to protect and help individuals maintain health and make necessary changes in lifestyle (Hall, 2013; Taylor et al., 2011). Previous studies have revealed that providing education to patients with CAD improves self-care agency (Hassani et al., 2010), quality of life (Küçükberber et al., 2011), and self-care information as well as motivation and skill levels (Mohammadpour et al., 2015).

In nursing practice, the use of theory helps systematize care planning, organizes professional knowledge into a conceptual framework, and guides nurses on how and why certain steps must be taken, thereby increasing the effectiveness of services by providing cost-effective care (Johnson, 2015). A crucial issue in improving quality of life in patients with chronic illness is ensuring patient participation in their treatment and care (Johnson, 2015). In this context, a basic principle of self-care is patient participation and assumption of responsibility (Hassani et al., 2010). Self-care, defined as performing individual duties to protect life, health, and well-being, develops gradually through communication, culture, education, and interaction (Berbiglia & Banfield, 2014; Fawcett & DeSanto-Madeya, 2013; Johnson, 2015). Orem's self-care deficit nursing theory (SCDNT) considers each individual as a self-care agent with the necessary ability to perform self-care activities individually (Berbiglia & Banfield, 2014; Fawcett & DeSanto-Madeya, 2013; Johnson, 2015).

In promoting the health status of patients with CAD, it is critical to effectively combat interchangeable risk factors, increase awareness, and improve self-care agency and quality of life (Butcher & Castelluci, 2011; Hassani et al., 2010). Therefore, training that is based on Orem's SCDNT has been hypothesized as an effective approach to preventing disease progression, reducing recurrent hospitalizations, minimizing financial expenses, improving self-care agency and quality of life, encouraging patient education, protecting health, and providing behavioral change by raising awareness toward development and teaching home care methods. This study was designed to determine the impact of a training program based on Orem's SCDNT on self-care agency and quality of life in patients with CAD.

## Methods

**Figure 1:**
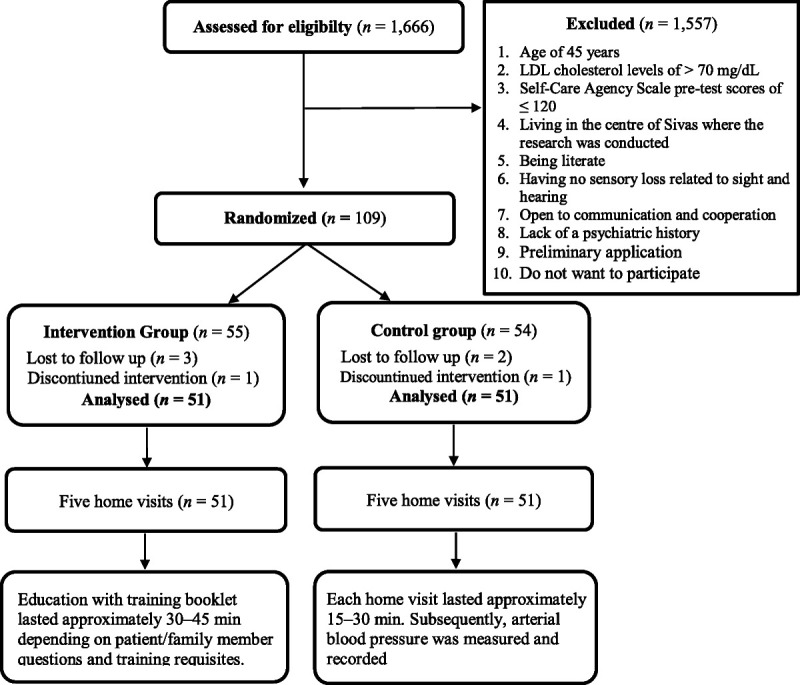
Flow diagram

This randomized, controlled, pretest/posttest experimental study was conducted between January 2015 and February 2017. The study population comprised patients with CAD from a cardiology clinic of a university and public hospital. One hundred two patients, 51 of whom were assigned to the intervention group and 51 of whom were assigned to the control group, were randomized according to gender, age (unmodifiable risk factors that increase the risk of CAD), low-density lipoprotein (LDL) cholesterol level (≥ 70 mg/dl; modifiable risk factors that increase the risk of CAD), and Self-Care Agency Scale score (≥ 120 points; Figure 1). The sampling criteria were as follows: (a) age of ≥ 45 years, (b) LDL cholesterol levels of > 70 mg/dl, (c) Self-Care Agency Scale pretest scores of ≤ 120, (d) living in the center of Sivas where the research was conducted, (e) being literate, (f) having no sensory loss related to sight or hearing, (g) being open to communication and cooperation, and (h) lack of a psychiatric history.

The study data were collected using the patient information form, Self-Care Agency Scale, MacNew Heart Disease Health-Related Quality of Life Questionnaire (MacNew), and Quality of Life Questionnaire (15D) during the second and third interviews, which were conducted, respectively, before hospital discharge and during the outpatient visit conducted 5–6 months after discharge.

### Patient Information Form

This form comprised 26 questions, including concomitant diseases, medications used, and general information, which were used to determine the risk factors for CAD (Butcher & Castelluci, 2011; Durmaz et al., 2009; Lukkarinen & Hentinen, 2006).

### Self-Care Agency Scale

The Self-Care Agency Scale, developed by Kearney and Fleischer (1979), with its validity and reliability in Turkish reported by Nahcivan (1993) in healthy young individuals and by Pınar (1995) in chronic diseases, has been adapted for use in Turkish settings. In this study, the version of the scale adapted by Pınar was used (Pınar, 1995). Validity and reliability study of the scale for use on patients with chronic diseases revealed a test–retest reliability of .80 and an internal consistency of .89 (Pınar, 1995). Furthermore, the pretest and posttest values of Cronbach's alpha reliability coefficient were determined to be .89 and .93, respectively. The Self-Care Agency Scale has been used to determine the self-care abilities of individuals, with high total scores indicating a high level of independence and abilities in achieving self-care. There are 35 items in the Turkish form, with each item evaluated using a score ranging from 0 to 4: 0 = *does not describe me at all*; 1 = *does not describe me very well*, 2 = *no idea*, 3 = *describes me a little*, and 4 = *describes me very well*. Eight of the scale items (3, 6, 9, 13, 19, 22, 26, and 31) are negatively stated, so the scoring method is reversed. The evaluation is based on 140 points, with a score below 82 points considered low, 82–120 points considered medium, and above 120 points considered high (Pınar, 1995).

### MacNew

MacNew, developed by the MacNew Group (MacNew, 2014), was tested for validity and reliability in Turkish and adapted to Turkish society by Daskapan et al. (2008). MacNew was designed to measure heart-disease-specific quality of life (MacNew, 2014). MacNew is a valid and easily applicable scale for patients with myocardial infarction, angina pectoris, or heart failure. There are strong correlations between MacNew and other quality-of-life scales, suggesting that MacNew is a valid and reliable quality-of-life measurement tool for patients with CAD (MacNew, 2014). In Daskapan et al., the Cronbach's alpha reliability coefficient of the questionnaire was determined to be .89. In this study, the pretest and posttest values of the Cronbach's alpha reliability coefficient for MacNew were .95 and .96, respectively. MacNew includes 27 items, each of which is scored using a 7-point Likert-type response, that are grouped into three subdimensions (emotional, physical, and social) as well as assessed as a total score. After assessing subdimensions, the averages of the items in each dimension are used. Therefore, the average scores vary from 1 to 7, with lower scores indicating worse quality of life and higher scores indicating better quality of life (MacNew, 2014). Only the total score for MacNew was used in this study.

### Quality of Life Questionnaire (15D)

15D, developed by Sintonen (2009) and tested for validity and reliability in Turkish by Akinci et al. (2005), has been adapted to Turkish settings to measure overall quality of life. The Cronbach's alpha reliability coefficient of 15D was found to be .99 in Akinci et al. (2005), and the pretest and posttest values were determined to be .62 and .83, respectively, in this study. 15D items are designed to assess movement, vision, hearing, breathing, sleeping, eating, speaking, excretion, normal activities, mental function, discomfort, depression, distress, vitality, and sexual activity. Each item offers five choices and is scored as 1 point. In 15D, individuals select one option, and scores are calculated. The first choice indicates the highest level, whereas the fifth choice indicates the lowest level. The obtained score is converted to a total score between 1 and 0, which indicates quality of life in terms of subjective health (1 = *quality of life with best health*, 0 = *quality of life with worst health*; Lukkarinen & Hentinen, 2006; Sintonen, 2009).

### Training Booklet

CAD-specific information was integrated into a training booklet in accordance with Orem's SCDNT by conducting a literature review and canvassing the opinions of related experts. Information on CAD was structured into three parts in the training booklet, covering the requisites of universal self-care, developmental self-care, and health deviation self-care. Universal self-care requisites include information on CAD such as maintaining adequate respiration, sustaining adequate fluid intake, maintaining adequate nutrient intake, maintaining adequate excretion, maintaining exercise–rest balance, maintaining balance between loneliness and the social environment, having protection from dangers that affect life and well-being, and being able to perform normal functions. Developmental self-care requisites include information on identifying and managing high-risk individuals. Finally, health deviation self-care requisites address the following topics: What is the responsibility of the heart? What is CAD? What are the risk factors for CAD? Which are the unmodifiable risk factors? Which are the modifiable risk factors? What should be done to achieve quality life with CAD? How can serum lipid level, hypertension, obesity, diabetes mellitus, stress, behavior patterns, and homocysteine level be controlled? How can one make lifestyle changes related to physical inactivity? What should be considered in drug treatment?

Interviews were conducted after completion of the 6-month postdischarge monitoring of behavioral changes and support provision. The participants were informed that they could use the training booklet as a guide but that they should not use the booklet as a substitute for referral/treatment by a physician. Furthermore, the participants were advised that they could call the researcher if they had any questions or concerns.

### Ethical Consideration

Before implementing this study, approval from the Faculty of Health Science Clinical Studies Ethics Committee of Erzurum Atatürk University (resolution number: 10/12/2014) was obtained and written permission from Sivas Cumhuriyet University, Healthcare Research Hospital, and Sivas Public Hospital was received. Written informed consent was obtained from each patient who met the inclusion criteria. The patients were informed that their participation in this study was their choice, that their names would be written on data-gathering forms, and that all personal information would be kept confidential. No intervention was included in the routine treatment of the two groups.

### Nursing Practice

All of the interviews were performed by the researcher F. T. Y. The research data from the preliminary practice were collected via face-to-face interviews from the patients who agreed to participate.

In the intervention group, data were collected during two sessions: at the hospital before discharge and at home after discharge. On average, 8–10 phone calls were made by the researcher to each of the participants or their relatives to plan home visits or to address questions/concerns.

Interview 1 was conducted at the cardiology clinic when hemodynamic indicators were normal on the first or second day after discharge from the intensive care unit. The participant was informed about the study, and verbal and written informed consents were obtained.

Interview 2 was conducted a day after the first interview at the cardiology clinic. The Self-Care Agency Scale was implemented, and LDL cholesterol level was recorded. The patients were randomized into an intervention group and a control group using the simple random sampling method based on age, gender, Self-Care Agency Scale score, and LDL cholesterol level.

Interview 3 was held a day after the second interview at the cardiology clinic. The pretest measurements (patient information form, MacNew, and 15D) were filled out by the researcher, and the participants' routine triglyceride–total cholesterol–HDL (high-density lipoprotein) cholesterol levels and arterial blood pressure were recorded at the hospital. The training was held in a quiet and calm private room with normal lighting and temperature to ensure comfort. Before the training, the patients were informed about the aim and goal of the training, including that the training would last approximately 40–50 minutes, that it would be interrupted and paused if necessary, and that questions could be asked whenever desired. The training booklet prepared to support learning with written and visual materials was given to participants, and the training content was projected on a projection screen.

Interview 4 was conducted at home after hospital discharge. At the first home visit, arterial blood pressure was measured and recorded.

Interview 5 was held at home within 3 days after the outpatient checkup in the first month. At the second home visit, arterial blood pressure was measured and recorded.

Interview 6 was held at home 4 weeks after the fifth interview. At the third home visit, arterial blood pressure was measured and recorded.

Interview 7 was held at home 3 days after the outpatient checkup in the third month. At the fourth home visit, arterial blood pressure, body mass index (BMI), and waist circumference were measured. The patients' triglyceride, total cholesterol, HDL cholesterol, and LDL cholesterol levels, which were routinely measured at the hospital, were recorded. The initial levels of modifiable risk factors were compared with the present levels. Changes that positively or negatively affected CAD were determined.

Interview 8 was conducted at home within 3 days after the outpatient checkup in the sixth month. At the fifth home visit, the patients' posttest measurements such as arterial blood pressure, BMI, and waist circumference were measured, and triglyceride–total cholesterol–HDL cholesterol–LDL cholesterol levels, which were routinely measured at the hospital during the outpatient visit in the sixth month, were recorded. The Self-Care Agency Scale, MacNew, and 15D were then filled out by F. T. Y.

In the intervention group, each home visit, conducted after hospital discharge, lasted approximately 30–45 minutes depending on each patient's questions and training requirements. The researcher asked each participant 12 questions that were prepared based on the content of the training, and behavioral changes were investigated. If deemed necessary, training was again provided to the participant during the interview.

In the control group, data were collected from two sessions, including that conducted before hospital discharge (Interview 1, Interview 2, and Interview 3) and after discharge. The pretest measurements, including the patient information form, MacNew, and 15D, were filled out by F. T. Y., and the participants' routine triglyceride–total cholesterol–HDL cholesterol levels and arterial blood pressure were recorded at the hospital during Interview 3. Each home visit after discharge (Interview 4, Interview 5, Interview 6, Interview 7, and Interview 8) lasted approximately 15–30 minutes. Subsequently, arterial blood pressure was measured and recorded. Counseling sessions on CAD for patients or their relatives were provided when deemed necessary. In addition, 8–10 phone calls were made to plan home visits and/or provide counseling to each patient or his or her relatives. In Interview 8, the patients' posttest measurements, including arterial blood pressure, BMI, and waist circumference, were measured and recorded. Triglyceride–total cholesterol–HDL cholesterol–LDL cholesterol levels, which were routinely measured at the hospital during the outpatient visit in the sixth month, were recorded, and the Self-Care Agency Scale, MacNew, and 15D were filled out by F. T. Y. Training was conducted, and the training booklet was provided to the control group patients at the end of the interviews (Interview 8).

### Evaluation of Data

Analyses of the study data were performed using IBM SPSS Statistics Version 20.0 (IBM, Inc., Armonk, NY, USA), and tables were created. Statistical results were presented as frequency and percentage based on the distribution of group characteristics related to the descriptive characteristics and CAD risk factors of the study groups. Chi-square analysis was performed to evaluate the characteristic distribution of the patients in the study groups with respect to descriptive characteristics and CAD risk factors and percentage distributions. Chi-square analysis was used for the homogeneity test, the Kolmogorov–Smirnov test was used to determine whether the pretest data were suitable for normal distribution to select the appropriate statistical analyses, and the Levene's test was used to determine the homogeneity of variances. Nonparametric tests were applied in all analyses because the normality analysis and the homogeneity of variance test did not show normal distribution. The Mann–Whitney *U* test was used for intergroup comparisons of the pretest and posttest values of triglyceride, total cholesterol, HDL cholesterol, LDL cholesterol, arterial blood pressure, BMI, waist circumference, and Self-Care Agency Scale, MacNew, and 15D scores. A Wilcoxon's signed-rank test was used for intragroup comparisons. Correlation analysis was used to determine the relationship between the average pretest and posttest scores on the Self-Care Agency Scale, MacNew, and 15D measures. Correlation coefficients of .00–.30 were considered low, .30–.70 were considered medium, and .70–1 were considered high (Büyüköztürk, 2010).

## Results

The distribution and comparison of the descriptive characteristics of the patients with CAD who participated in this study are summarized in Table 1. No significant difference was observed between the groups in terms of descriptive characteristics, and the groups were homogeneous in terms of the study variables (*p* > .05).

**Table 1:** Distribution and Comparison of Patients by Descriptive Characteristics

Characteristic	Intervention Group (*n* = 51)		Control Group (*n* = 51)		χ^2^	*p*
	*n*	%	*n*	%		
Age (years; *M* and *SD*)	59.98	7.42	56.74	7.55	5.76	.21
45–49	4	7.8	6	11.8		
50–54	9	17.7	18	35.3		
55–59	12	23.5	10	19.6		
60–64	16	31.4	9	17.6		
65 and above	10	19.6	8	15.7		
Gender					0.92	.33
Female	42	82.4	38	74.5		
Male	9	17.6	13	25.5		
Marital status					1.38	.24
Single	5	9.8	2	3.9		
Married	46	90.2	49	96.1		
Educational level					7.74	.17
Literate	3	5.9	2	3.9		
Primary	31	60.8	33	64.7		
Secondary	2	3.9	5	9.8		
High school	9	17.6	11	21.6		
University	5	9.8	–	–		
Master/doctorate	1	2.0	–	–		
Occupation					4.94	.29
Retired	32	62.7	29	56.9		
Housewife	7	13.7	13	25.5		
Civil servant	7	13.7	3	5.9		
Other	5	9.9	6	11.8		
Income level					0.67	.71
Lower than expenses	46	90.2	13	25.5		
Balanced	–	–	35	68.6		
Higher than expenses	5	9.8	3	5.9		
Lifestyle					2.53	.86
Single	2	3.9	2	3.9		
With spouse	12	23.5	10	19.6		
With spouse and children	34	66.7	35	68.6		
Other	3	5.9	4	7.9		
Diagnosis					0.40	.52
Angina pectoris	15	29.4	18	35.3		
Myocardial infarction	36	70.6	33	64.7		
Chronic disease					0.40	.52
Yes	33	64.7	36	70.6		
No	18	35.3	15	29.4		

Regarding hereditary CAD, serum lipid level, BMI, waist circumference, hypertension, diabetes, smoking, alcohol, sedentary lifestyle, and stressors related to CAD risk factors, no significant difference was identified between the two groups (*p* > .05). In addition, the groups had similar characteristics in terms of the study variables related to CAD risk factors. In both groups, the pretest values for LDL cholesterol level, total cholesterol level, triglyceride level, and waist circumference were high. Furthermore, the posttest LDL cholesterol and total cholesterol levels of the two groups showed lower intragroup average scores, with a highly significant intergroup difference (*p* < .01; Table 2).

**Table 2:** Distribution and Comparison of Patients by CAD Risk Factors

Distribution	Intervention Group (*n* = 51)		Control Group (*n* = 51)		χ^2^	*p*
	*n*	%	*n*	%		
Hereditary					0.20	.65
Yes	37	72.5	39	76.5		
No	14	27.5	12	25.5		
Serum lipids: LDL cholesterol (mg/dl; posttest)^a^					0.63	.42
≤ 70	7	13.7	10	19.6		
> 70	44	86.3	41	80.4		
Total cholesterol (mg/dl; pretest)					0.99	.31
≤ 200	31	60.8	26	51.0		
> 200	20	39.2	25	49.0		
Total cholesterol (mg/dl; posttest)					0.82	.36
≤ 200	36	70.6	40	78.4		
> 200	15	29.4	11	21.6		
Triglyceride (mg/dl; pretest)					0.98	.32
≤ 150	23	45.1	28	54.9		
> 150	28	54.9	23	45.1		
Triglyceride (mg/dl; posttest)					0.35	.55
≤ 150	30	58.8	27	52.9		
> 150	21	41.2	24	47.1		
HDL cholesterol (mg/dl; pretest)					0.36	.54
≥ 40	23	45.1	20	39.2		
< 40	28	54.9	31	60.8		
HDL cholesterol (mg/dl; posttest)					0.62	.42
≥ 40	23	45.1	27	52.9		
< 40	28	54.9	24	47.1		
BMI (kg/m^2^; pretest)					0.36	.54
≤ 30	32	62.7	29	56.9		
> 30	19	37.3	22	43.1		
BMI (kg/m^2^; posttest)					1.02	.31
≤ 30	33	64.7	28	54.9		
> 30	18	35.3	23	45.1		
Waist circumference (cm; pretest)					0.05	.81
♀: ≤ 88, ♂: ≤ 95	12	23.5	13	25.5		
♀: ≥ 88, ♂: ≥ 95	39	76.5	38	74.5		
Waist circumference (cm; posttest)					0.92	.33
♀: ≤ 88, ♂: ≤ 95	13	25.5	9	17.6		
♀: ≥ 88, ♂: ≥ 95	38	74.5	42	82.4		
Hypertension^b^					2.54	.11
Yes	26	78.8	22	61.1		
No	7	21.2	14	38.9		
Diabetes^b^					0.16	.68
Yes	14	42.4	17	47.2		
No	19	57.6	19	52.8		
Cigarette					1.32	.51
Smoking	21	41.2	24	47.1		
No smoking	15	29.4	17	33.3		
Quit smoking	15	29.4	10	19.6		
Alcohol					0.37	.82
Drinking	1	2.0	2	3.9		
No drinking	38	74.5	38	74.5		
Quit drinking	12	23.5	11	21.6		
Sedentary lifestyle					2.13	.14
Yes	14	27.5	21	41.2		
No	37	72.5	30	58.8		
Stressors					0.56	.81
Yes	40	78.4	39	76.5		
No	11	21.6	12	23.5		

*Note.* CAD = coronary artery disease; BMI = body mass index; HDL = high-density lipoprotein; LDL = low-density lipoprotein.

^a^LDL cholesterol pretest measurement of >70 mg/dl is the sampling criterion and so is not included in the table.

^b^Numbers of patients with chronic diseases: intervention group, *n* = 33; control group, *n* = 36.

When the average pretest and posttest scores on the Self-Care Agency Scale were compared, the average intragroup scores of the intervention group were found to be significantly higher than those of the control group (*p* < .01). In addition, there was a significantly greater increase in the last average score of the control group than that of the intervention group (*p* > .05). However, the average posttest scores on the Self-Care Agency Scale were significantly higher in the intervention group than in the control group (*p* < .01; Table 3).

**Table 3:** Comparison of Pretest and Posttest Mean Scores of Self-Care Agency Scale, MacNew, and 15D

Scale	Intervention Group			Control Group			*U*	*p*
	*M*	*SD*	Median	*M*	*SD*	Median		
Self-Care Agency Scale								
Pretest	93.09	18.30	96.00	87.68	23.44	87.00	1110.0	.20
Posttest	119.96	12.27	121.00	91.23	21.04	92.00	287.5	< .001
	*z* = −5.923, *p* < .001			*z* = −1.275, *p* = .20				
MacNew								
Pretest	5.09	1.36	5.40	5.03	1.38	5.07	1270.5	.84
Posttest	6.40	0.61	6.59	5.01	1.10	5.25	271.0	< .001
	*z* = −5.672, *p***<** .001			*z* = −0.145, *p* = .88				
15D								
Pretest	0.85	0.06	0.84	0.85	0.07	0.85	1279.0	.88
Posttest	0.95	0.05	0.96	0.86	0.08	0.85	455.0	< .001
	*z* = −5.952, *p* < .001			*z* = −0.994, *p* = .32				

*Note.* MacNew = MacNew Heart Disease Health-Related Quality of Life Questionnaire; *U* = Mann–Whitney *U* test; *z* = Wilcoxon sign test; 15D = Quality of Life Questionnaire.

Table 3 shows a comparison of the average pretest and posttest scores on MacNew. According to the pretest intragroup scores on this measure, the average posttest scores were significantly higher in the intervention group than in the control group (*p* < .01), which recorded a decline in average posttest scores (*p* > .05). In addition, the average intergroup posttest scores on MacNew were significantly higher in the intervention group than in the control group (*p* < .01).

With regard to the average pretest and posttest scores on 15D (Table 3), the intervention group showed an increase in average scores (*p* < .01), and in the intergroup analysis, the average posttest score was significantly higher in the intervention group than in the control group (*p* < .01).

A statistically significant correlation was found at the low–medium level on the positive side, ranging from .288 to .587 between the average pretest and posttest scores on the Self-Care Agency Scale and MacNew in both groups. Moreover, a statistically significant correlation was found at the low–medium level on the positive side, ranging from .369 to .482 between the average pretest and posttest scores on the Self-Care Agency Scale and 15D in both groups. Furthermore, a statistically significant correlation was noted at the medium–high level on the positive side, ranging from .677 to .852 on MacNew and 15D in both groups.

## Discussion

When the distribution according to CAD risk factors was examined, it was found that both groups widely shared family histories of CAD and lived a sedentary lifestyle and stressful lives. The posttest LDL cholesterol level was found to be higher than the pretest level in both groups. Similarly, previous studies have found that individuals with CAD tend to have a family history of the disease (Herman et al., 2014; Kang & Yang, 2013; Saffi et al., 2014), to live a sedentary lifestyle (Saffi et al., 2014; Tokgözoğlu et al., 2010), and to have stressful lives (Herman et al., 2014). In this study, the two groups were distributed homogeneously based on CAD risk factors, with results consistent with results previously reported in the literature. Steps toward identifying effective risk factors in CAD formation, providing primary protection to the population and to high-risk individuals, providing secondary protection through the identification of the current risk factors for CAD, and improving quality of life and tertiary protection, including morbidity and mortality rate reduction and rehabilitation implementation, have gained in importance (Onat, 2009; WHO, 2019). In this respect, the fact that the risk factors of CAD were identified in this study as well as in the control group patients is considered to be an indicator that the patient sample was properly selected.

In both groups, only the average posttest LDL cholesterol and total cholesterol levels were low, and the intragroup difference was statistically significant. Studies have reported a decrease in intergroup levels of LDL cholesterol (Jiang et al., 2007; Saffi et al., 2014; Wood et al., 2008), triglyceride (Jiang et al., 2007), and total cholesterol (Jiang et al., 2007; Wood et al., 2008), whereas intergroup differences have been found to be highly significant only in these studies (Jiang et al., 2007; Wood et al., 2008). The training provided by a researcher on cholesterol-lowering drug therapy and dietary and lifestyle changes may account for the decrease in the LDL cholesterol and total cholesterol levels in the intervention group. The decrease in LDL cholesterol and total cholesterol levels in the control group may be associated with the recommendations of cardiologists and nurses regarding cholesterol-lowering drug treatments and dietary and lifestyle changes given before discharge and during outpatient clinic visits, as no statistically significant differences were observed between the average intergroup pretest and posttest scores for serum lipid levels, arterial blood pressure, and waist circumference.

The average intragroup and intergroup posttest scores for the intervention group on the Self-Care Agency Scale were found to be significantly higher than those for the control group (Table 3). In a prior study conducted to determine self-care agency in patients with CAD, self-care agency (59.13 ± 12.62) was found to be low (Hassani et al., 2010). In this study, the higher average self-care agency score and better self-care agency in the intervention group than in the control group are consistent with the findings previously reported in the literature. Therefore, the training program based on Orem's SCDNT increased the level of CAD-related awareness and improved lifestyle habits and disease risk factors. Furthermore, the intervention was effective in improving self-care agency by increasing awareness of, ability to utilize, and ability to further enhance their self-care agency.

In addition, the average posttest scores on MacNew for the intervention group were significantly higher than those for the control group (Table 3). In studies using MacNew to assess quality of life in patients with cardiac disease (Daskapan et al., 2008; Höfer et al., 2012; Sevinç & Akyol, 2010), good results have been reported. In comparing the results of this study with those in the literature (Daskapan et al., 2008; Höfer et al., 2012; Sevinç & Akyol, 2010), the average quality-of-life score related to CAD was found to be higher in the intervention group than in the control group. This result suggests that the training based on Orem's SCDNT resulted in positive behavioral changes in the intervention group that allowed participants to better manage their disease.

Table 3 shows the comparison between average pretest and posttest scores on 15D. In the intervention group, the difference between the average intragroup and intergroup posttest scores on 15D was significantly larger than that in the control group. In previous studies that used 15D (Heiskanen et al., 2016; Sintonen, 2009), patients were found to have a good quality of life (mean score: 0.82–0.95). The overall quality of life of the intervention group in this study seems to be better than that of patients in other studies (Heiskanen et al., 2016; Sintonen, 2009). This result may relate to the fact that the training based on Orem's SCDNT improved self-care agency and cardiac-disease-specific quality of life by effectively promoting lifestyle changes. The nursing initiative that was employed to improve self-care skills in the intervention group may also have improved self-care abilities and disease-related adjustments, while also boosting overall quality of life by improving functional abilities and promoting the effective management of disease progression.

To the best of our knowledge, no previous study has evaluated the correlation among the Self-Care Agency Scale, MacNew, and 15D in patients with CAD. However, several studies have evaluated the correlation between various scales such as the Self-Care Agency Scale, the Short Form-36, EuroQoL-5 dimension, 15D, and MacNew in chronic diseases such as CAD, chronic obstructive pulmonary disease, and diabetes (Heiskanen et al., 2016; Höfer et al., 2008). In these studies, self-care agency and disease-specific and overall quality of life were found to affect each other at low (Heiskanen et al., 2016), middle (Höfer et al., 2008), and high (Akinci et al., 2005) levels. Therefore, determining self-care agency and the disease-specific and overall quality of life of patients with CAD positively contributes to current scholarly knowledge on this subject.

This study is affected by several limitations. Although CAD is among the most frequently observed diseases and a common cause of death worldwide, the number of patients included in this study was limited (102). In addition, conducting the last follow-up during the autumn–winter season limited this study to home environments and ended the measurement of physiological parameters (BMI and waist circumference) in early autumn.

In conclusion, the Orem's SCDNT-based training program used in this study significantly increased self-care agency and disease-specific and overall quality of life in the intervention group. The use of theory in nursing practice systematizes care planning and organizes professional knowledge into a conceptual framework, providing an effective guide to nurses in terms of what to do and why actions should be taken. In addition, nurses should pay closer attention to the CAD-related educational level of their patients with CAD to best teach them how to live with their disease. Therefore, it is suggested that Orem's SCDNT be used as a guide to strengthen self-care and increase quality of life in patients with CAD from the time of hospital admission and discharge through the postdischarge period. Furthermore, medical institutions and governments should develop educational policies for patients at risk of CAD and for those with CAD.
